# Long-term exposure to fine particulate matter and incidence of type 2 diabetes mellitus in a cohort study: effects of total and traffic-specific air pollution

**DOI:** 10.1186/s12940-015-0031-x

**Published:** 2015-06-19

**Authors:** Gudrun Weinmayr, Frauke Hennig, Kateryna Fuks, Michael Nonnemacher, Hermann Jakobs, Stefan Möhlenkamp, Raimund Erbel, Karl-Heinz Jöckel, Barbara Hoffmann, Susanne Moebus, on behalf of the Heinz Nixdorf Recall Investigator Group

**Affiliations:** IUF - Leibniz Research Institute for Environmental Medicine, Düsseldorf, Germany; Medical School, Heinrich Heine University of Düsseldorf, Düsseldorf, Germany; Institute for Medical Informatics, Biometry and Epidemiology, University Hospital of Essen, University of Duisburg-Essen, Essen, Germany; Rhenish Institute for Environmental Research at the University of Cologne, Cologne, Germany; Department of Cardiology, West German Heart Centre of Essen, University of Duisburg-Essen, Essen, Germany

**Keywords:** Type 2 diabetes, Particulate matter, PM_10_, PM_2.5_, Air pollution, Traffic

## Abstract

**Background:**

Studies investigating the link between long-term exposure to air pollution and incidence of diabetes are still scarce and results are inconsistent, possibly due to different compositions of the particle mixture. We investigate the long-term effect of traffic-specific and total particulate matter (PM) and road proximity on cumulative incidence of diabetes mellitus (mainly type 2) in a large German cohort.

**Methods:**

We followed prospectively 3607 individuals without diabetes at baseline (2000–2003) from the Heinz Nixdorf Recall Study in Germany (mean follow-up time 5.1 years). Mean annual exposures to total as well as traffic-specific PM_10_ and PM_2.5_ at residence were estimated using a chemistry transport model (EURAD, 1 km^2^ resolution). Effect estimates for an increase of 1 μg/m^3^ in PM were obtained with Poisson regression adjusting for sex, age, body mass index, lifestyle factors, area-level and individual-level socio-economic status, and city.

**Results:**

331 incident cases developed. Adjusted RRs for total PM_10_ and PM_2.5_ were 1.05 (95 %-CI: 1.00;1.10) and 1.03 (95 %-CI: 0.95;1.12), respectively. Markedly higher point estimates were found for local traffic-specific PM with RRs of 1.36 (95 %-CI: 0.98;1.89) for PM_10_ and 1.36 (95 %-CI: 0.97;1.89) for PM_2.5_. Individuals living closer than 100 m to a busy road had a more than 30 % higher risk (1.37;95 %-CI: 1.04;1.81) than those living further than 200 m away.

**Conclusions:**

Long-term exposure to total PM increases type two diabetes risk in the general population, as does living close to a major road. Local traffic-specific PM was related to higher risks for type two diabetes than total PM.

**Electronic supplementary material:**

The online version of this article (doi:10.1186/s12940-015-0031-x) contains supplementary material, which is available to authorized users.

## Background

High levels of particulate matter (PM) have consistently been shown to increase mortality and morbidity worldwide [1, 2]. Most evidence relates to short-term and long-term effects on cardiopulmonary disease [2, 3]. In several studies, diabetic subjects have been reported to be an especially susceptible group for air pollution related cardiovascular health effects compared to the general population [4–7]. However, while diabetic patients seem to constitute a vulnerable group for air pollution effects, much less is known as to whether air pollution also plays a role in pathogenesis and incidence of diabetes itself.

A positive association between diabetes and air pollution was first observed in ecological comparisons [8] followed by cross-sectional studies [9–11], but see [12] for a different result. A recent investigation based on the large Danish Diet, Cancer, and Health cohort (DDCH) observed an association with diabetes-related mortality [13]. Only five prospective studies on diabetes incidence investigating six cohorts, three women cohorts among them, have been published [14–18]. While all of these studies that investigated NO_2_ found an effect on diabetes incidence, the results for PM were less clear. The results of these studies have been reviewed and synthesized recently [19–21]. Even more recently, a study from the US showed an association of PM2.5 and NO_2_ with diabetes prevalence but not incidence [22].

The mechanisms by which particulate matter may lead to diabetes are still unclear and an area of active research. One main working hypothesis is that PM, by inducing oxidative stress and subsequent systemic inflammation [3], leads to increased insulin resistance. A link of PM_10_ with systemic inflammation has been shown in several studies (see e.g., [3] for a review) and in our own cohort [23, 24].

The effects of PM on health outcomes are hypothesized to depend on its constituents and their toxicity [25]. Several studies have indicated that particularly traffic-related air pollution with its primary combustion particles has important health effects ([25] and references therein) and causes a substantial part of the health impact related to ambient air pollution [26].

In the present study, we investigate the influence of total PM and PM emitted from local traffic on the cumulative incidence of type 2 diabetes in a highly urbanized region of Germany. The analysis is based on the well-characterized population-based Heinz Nixdorf Recall Study. We apply for the first time a source-specific dispersion and chemistry transport model to assess total and traffic-specific PM exposure.

## Methods

### The Heinz Nixdorf Recall study

The ongoing Heinz Nixdorf Recall (**R**isk Factors, **E**valuation of **C**oronary **Cal**cium and **L**ifestyle) study investigates a population-based random sample of men and women living in three adjacent cities of the densely populated Ruhr Area in Germany. The rationale and design of the study have been described in detail [27]. Briefly, an age-stratified random sample of individuals aged 45–75 years was drawn from mandatory lists of residents of Essen, Bochum and Mülheim. 4,814 participants were recruited between 2000 and 2003 corresponding to a participation rate of 56 %. The study was approved by the local ethics committees and includes extended quality management procedures. Informed consent was obtained from all participants. The baseline assessment included a self-administered questionnaire and face-to-face interviews for life-style and personal risk factor assessment (including smoking status, alcohol intake, education, occupational status and physical activity), a clinical examination including blood pressure measurements, anthropometric measurements and comprehensive clinical and laboratory tests according to standard protocols [27, 28]. A five-year follow up was conducted between 2006 and 2008 with identical assessment procedures.

### Outcome

Incident diabetes mellitus (DM) in those free of disease at baseline was defined as a self-reported physician diagnosis or incident intake of an anti-diabetic drug (ATC-code A10) during follow-up or random blood glucose ≥ 200 mg/dL or fasting blood glucose ≥ 126 mg/dL at the first follow-up examination (approximately 5 years after baseline). All subjects had glucose measurements at baseline and follow-up, approximately 70 % had fasting glucose. For those who did not have fasting glucose, in general, glucose levels were reduced quickly (2 h or less) after the last caloric intake [29]. In sensitivity analyses, we analyzed the effect of PM on “known DM”, defined as self-reported physician diagnosis or intake of an anti-diabetic drug (ATC-code A10), to prevent overdiagnosis of DM based purely on the blood glucose measurements on the day of the baseline and follow-up exam, respectively. Our method does not allow for the distinction of diabetes mellitus type 2 vs. type 1, but given the advanced age of our study participants, incident cases can be assumed to be mainly type 2 diabetes melllitus.

### Exposure

PM_10_ (aerodynamic diameter ≤ 10 μm) and PM_2.5_ (aerodynamic diameter ≤ 2.5 μm) concentrations were estimated with the European Air Pollution Dispersion and Chemistry Transport Model (EURAD-CTM) on a spatial resolution of 1 km^2^ grid cells. Details of the model have been described before [30, 31]. In short, the EURAD-CTM uses input data from official emission inventories on a scale of 1 km^2^, including industrial sources, household heating, traffic and agriculture and data on hourly meteorology and regional topography. Surface concentrations are calculated by dispersing emissions in horizontal strata, taking chemical reactivity and transport processes into account. Furthermore pollutants coming into the area by long-range transport are taken into account. The values obtained for total PM_10_ were compared to actual measurements from monitoring sites resulting in a calibration (data assimilation) of the EURAD model. This adapted model shows a good agreement between modelled values and actual measurements (correlation coefficient for daily means > 0.80). Modelled values were calculated for each 1 km^2^ grid cell within the study area and assigned to the residential addresses of the participants (ArcView 9.2). The model estimates daily mean concentrations. From these daily values the mean concentrations for longer time periods such as annual means etc. are calculated. The mean concentration of the years 2001 and 2002 was used to reflect the pattern of long-term residential exposure. We considered these two years to approximate best the long-term exposure because the year 2003 was very unusual regarding its meteorology, e.g., it was the year of a great heat wave in Europe.

Traffic-specific modelling was done by suppression of local traffic sources, in order to model concentrations of particulate matter and gaseous pollutants originating from all other sources except local traffic (PM_noTRA_). Subsequently, traffic-specific PM concentrations (PM_TRA_) were calculated by subtraction from total PM (PM_ALL_) as PM_TRA_ = PM_ALL_-PM_noTRA_.

As additional traffic exposure we used the distance to the next road with a traffic density higher than the 80 %-percentile (26062 vehicles/day) in the study region.

### Statistical analysis

Analyses were performed on the part of the population that did not have diabetes at baseline. Incident cases were assessed at the follow-up exam and cumulative incidence was calculated. At baseline and follow-up, the same diabetes definition was used. We used the Pearson correlation coefficient to examine the correlation between long-term total and traffic-specific PM concentrations. Because the exact date of the diabetes diagnosis is unknown, the association of diabetes incidence with air pollution was investigated using Poisson regression adapted to binary outcomes [32]. Based on prior biological and epidemiological knowledge, we specified potential confounders and correspondingly, in our main model, we adjusted for age, sex, smoking status, physical activity, BMI, individual socioeconomic status (SES), neighborhood unemployment rate and an indicator for city. Alcohol consumption and hypertension were also tested but did not influence the effect estimate and were therefore omitted. Continuous terms were introduced for age, BMI and BMI square (both centered on mean BMI) and neighborhood unemployment rate, while the other covariates were entered as categorical variables (Table 1). To allow comparisons of the effects of total vs. traffic-specific pollution, the respective effect estimates are also expressed per 1 μg/m^3^ increase in PM. In a sensitivity analysis of the traffic variables, we took into account the overall level of PM by 1) adjusting PM_TRA_ for PM_noTRA_ and 2) adjusting road proximity for PM_ALL_. To take into account very small scale differences in traffic exposure, which are not reflected in the EURAD model output, we also adjusted PM_TRA_ for road proximity and vice versa. Moreover, we conducted the regression analysis without the indicator for city: while the model with city reflects the effects of differences within city, the model without city refers to the overall contrasts in the whole study area, however potentially confounded by city-specific characteristics. Further sensitivity analyses included a restriction of the analysis to participants who did not relocate during follow up, the analysis of known diabetes as an alternative outcome and an adjustment for diabetogenic medication (i.e. neuroleptics, pentamidine, nicotinic acid, glucocorticoids, thyroideal hormones, diazoxides, ß-adrenergic agonists, thiazides, phenytoine and alpha-interferon).

**Table 1:** Baseline characteristics of the study population (N = 3607) stratified by diabetes status at follow up

	Participants without diabetes N = 3276	Participants with incident diabetes N = 331
Variable		
Men, %	47	56
Age [years], mean (SD)	58.8 (7.6)	60.5 (7.5)
BMI [kg/m^2^], mean (SD)	27.2 (4.2)	29.8 (4.7)
BMI > 25, %	69	87
BMI > 30, %	22	44
Hypertension^a^, %	50	66
Non-Smokers, %	44	39
Smokers, %	22	21
Ex-smokers, %	34	40
Exercise >3 times per week, %	26	22
% unemployment rate in neighbourhood mean (SD)	12.4 (3.4)	12.6 (3.4)
Mülheim, %	38	37
Essen, %	34	33
Bochum, %	29	31
Occupational status		
Employed, %	45	34
Inactive, %	15	13
Pensioner, %	35	45
Unemployed, %	6	8
Education ^b^		
Highest, %	13	6
High, %	23	21
Middle, %	55	63
Low, %	10	10
PM10 total [μg/m^3^], mean (SD)	20.8 (2.3)	20.9 (2.4)
PM2.5 total [μg/m^3^], mean (SD)	16.7 (1.4)	16.8 (1.5)
PM10 traffic [μg/m^3^], mean (SD)	0.8 (0.2)	0.9 (0.2)
PM2.5 traffic [μg/m^3^], mean (SD)	0.8 (0.2)	0.9 (0.2)
Distance to major road [m], mean (SD)	1021.4 (805.1)	1034.6 (830.5)

*N* number of individuals, *SD* standard deviation,

^a^Hypertension is defined as systolic blood pressure ≥ 140 mmHg or diastolic blood pressure *≥* 90 mmHg or intake of hypertensive medication

^b^Education: low: ≤10 years, middle 11–13 years, high: 14–17 years, highest:≥18 years

Effect modification was investigated by introducing the potential effect modifier as dichotomous variable and the corresponding multiplicative interaction term into the model. The following factors were investigated: age (<65, ≥ 65 years), sex, physical activity (<3, ≥3 times a week), BMI (<30 kg/m^2^, ≥ 30 kg/m^2^), education (≤13 years, >13 years), smoking status (smoker, non-smoker, ex-smoker) and hs-CRP (≤ the median of 0.1305 mg/dL, >0.1305 mg/dL).

## Results

At baseline, 4154 study participants had no diabetes. The follow-up population i.e., the population for which data from the follow-up exam were available (3640 participants) did not differ substantially from the original base population. The characteristics that differed most were being employed, being a pensioner and having hypertension with 41.6 %, 37.6 % and 53.6 %, at baseline vs. 43.6 %, 35.6 % and 51.6 %, at follow-up. Of those 3640 participants, 3607 had complete information on all covariates and represent the study population for our analysis. The mean follow-up time, i.e., the time between the first and the second visit, was 5.1 (SD 0.3; range 4.2 to 7.5) years.

331 individuals developed diabetes during follow-up. With respect to those who did not develop diabetes, these participants were slightly older, had a higher BMI and more frequently hypertension at baseline (Table 1). There were less non-smokers counterbalanced by more ex-smokers and a smaller proportion reported to exercise more than three times per week in the incident diabetic group. Also, participants developing diabetes were less often employed, more often pensioners, and had more often the middle educational level and less often the highest educational level. The distribution of incident cases among cities was very similar to the individuals that did not develop diabetes.

The mean exposure of the study population was 20.8 μg/m^3^ PM_10ALL_ (interquartile range (IQR) = 3.78 μg/m^3^) and 16.7 μg/m^3^ PM_2.5ALL_ (IQR = 2.29 μg/m^3^) (Table 2, Additional file 1: Table S1). Mean mass concentrations of local traffic-specific PM were considerably lower. The distance of individuals’ residence to a busy road (>26,062 vehicles/day) ranged from 1 m to 4877 m. Correlation of traffic-specific PM was moderate to low with total PM (Pearson correlation coefficient of 0.32 for PM_10_ and 0.39 for PM_2.5_), distance to a busy road (−0.45 for PM_10_ and PM_2.5_) and PM_noTRA_ (0.43 for PM_10_ and 0.23 for PM_2.5_).

**Table 2:** Association of total and traffic-specific pollutants and diabetes incidence (relative risks for PM are presented for an increase equivalent to the IQR and for an increase of 1 μg/m^3^)

			Increase in PM equivalent to the IQR		Increase of 1 μg/m^3^ PM	
		IQR	Crude model	Main model^a^	Crude model	Main model^a^
Total PM	PM_10ALL_	3.78	1.08 (0.96;1.21)	1.20 (1.01;1.42)	1.02 (0.99;1.05)	1.05 (1.00;1.10)
	PM_2.5All_	2.29	1.03 (0.92;1.15)	1.08 (0.89;1.29)	1.01 (0.96;1.06)	1.03 (0.95;1.12)
Traffic	PM_10TRA_	0.33	1.15 (1.05;1.27)	1.11 (0.99;1.23)	1.54 (1.15;2.05)	1.36 (0.98;1.89)
	PM_2.5TRA_	0.32	1.15 (1.04;1.26)	1.10 (0.99;1.23)	1.53 (1.15;2.05)	1.36 (0.97;1.89)
Distance to major road (>200 m reference) (N = 3186)	<= 100 (N = 180)				1.31 (0.99;1.75)	1.37 (1.04;1.81)
	>100-200 (N = 339)				0.81 (0.60;1.12)	0.77 (0.57; 1.04)

^a^main model adjusted for age, gender, lifestyle variables, BMI, individual and neighbourhood SES, and city

*N* numbers of individuals

The relative risks from the unadjusted crude model and for the main model are shown in Table 2. When expressing RRs per IQR, exposure to total PM_10_ was related to an increase in type 2 diabetes incidence of 20 % (RR of 1.20, 95 %-CI: 1.01;1.31) in the main model. The corresponding RR for PM_2.5_ was 1.11 (95 %-CI: 0.99;1.23). For traffic-specific PM, the estimates for this measure of population distribution of exposures were similar with a RR of 1.11 (95 %-CI: 0.99;1.17) for PM10_TRA_ and a RR of 1.10 (0.99;1.23) for PM2.5_TRA_.

However, when expressing the estimates for an increase of 1 μg/m^3^, RRs were 1.05 (95 %-CI: 1.00;1.10) for PM_10_ and 1.03 (95 %-CI: 0.95;1.12) for PM_2.5_. The estimated RRs for traffic-specific PM were now in comparison markedly higher than for total PM with RRs of 1.36 (0.98;1.89) for PM_10TRA_ and 1.36 (0.97;1.89) for PM_2.5TRA_. In comparison to subjects that lived more than 200 m away from busy roads, there was an increased risk of type 2 diabetes for those living within 100 m with a RR of 1.37 (95%CI: 1.04; 1.81), whereas no increased risk was observed for individuals living at intermediate distances (Table 2).

In sensitivity analyses, lack of adjustment for city reduced the effect size for PM_ALL_ but not for PM_TRA_ (see Additional file 1: Figure S1). In two-exposure-models, i.e., adjusting the model for PM_TRA_ with the mass of PM not attributable to traffic (PM_noTRA_), the effect estimate per 1 μg/m^3^ increase did not change for PM_2.5TRA_ (1.37; 95 %-CI: 0.96-1.93) but decreased for PM_10TRA_ (1.20; 95 %-CI: 0.82-1.76). All other sensitivity analyses did not change the results noticeably.

**Fig. 1:**
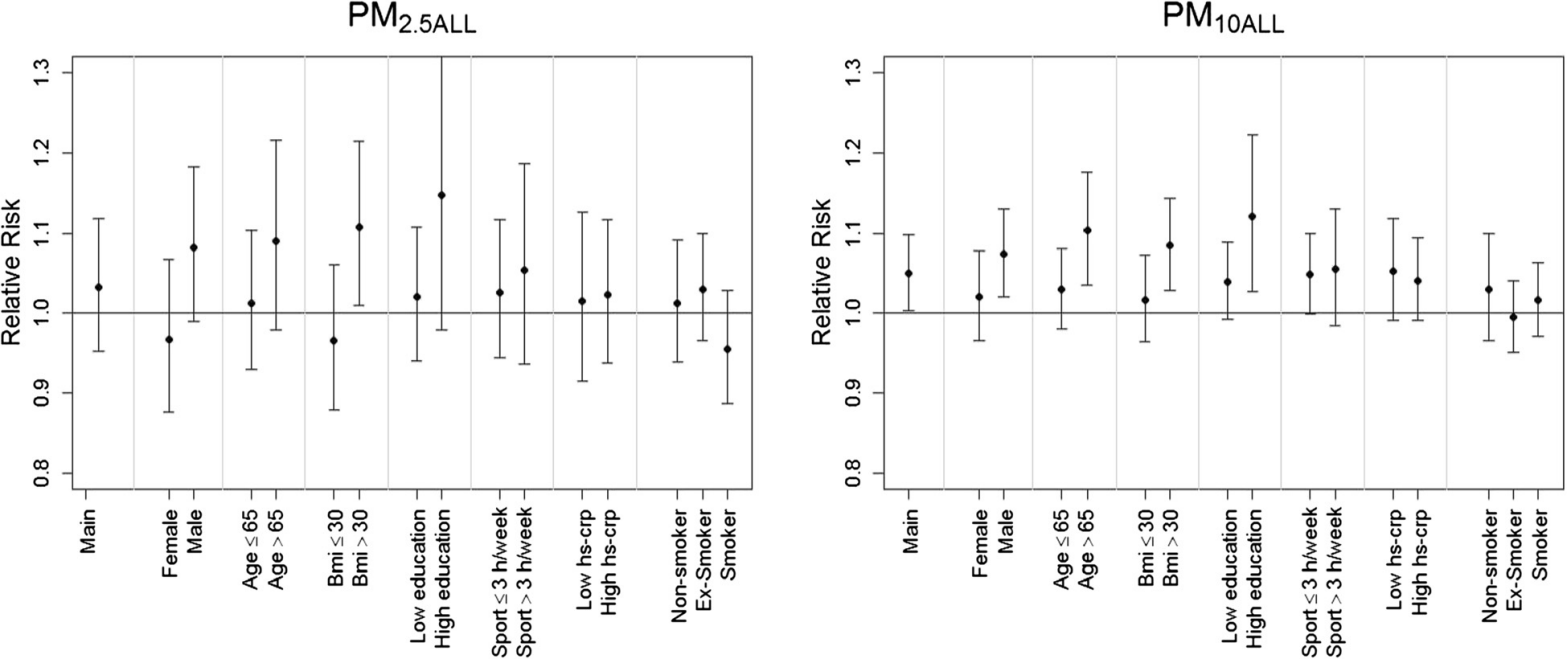
Effect modification of the effect of total PM: Relative risks (RR) with 95 % confidence intervals from the main model (i.e., adjusted for sex, age, BMI, lifestyle, individual SES, neighborhood unemployment rate and city)

Higher effect estimates were seen in men and in individuals with a BMI above 30 kg/m^2^ for total PM_10_ and PM_2.5_ (Fig. 1). We also observed higher effect estimates for individuals with high education and age ≥ 65 years or older. No differences in PM effect estimates were seen for degree of physical exercise, smoking status and hs-CRP. The pattern for effect modification was very similar for PM_TRA_ except for CRP where lower effect estimates were observed in individuals with CRP at or below the median.

## Discussion

Our study points to a possible effect of total PM and more specifically of PM related to local traffic on type 2 diabetes risk in the general population of a highly urbanized region in Germany. When the effects are compared per equal mass (1 μg/m^3^), PM related to local traffic shows an approximately 10 fold effect, or even stronger than that. The importance of traffic-specific PM is underscored by the fact that the effect size for living closer than 100 m to a busy road is similar to that for 1 μg/m^3^ traffic-specific PM. The effect per 1 μg/m^3^ total PM_10_ corresponds to an increase in risk related to two years of older age. It translates into a RR of 3.5 for the most exposed person vs. the least exposed person in our study.

Our results are stronger than the previously reported positive association of PM with diabetes incidence from the SALIA study in the same region, i.e., the Ruhr Area but based on different air pollution assessments and a different study population (elderly women only) [14]. Krämer et al. found a RR for self-reported diabetes of 1.15 for an IQR of 10.4 μg/m^3^ PM_10_ measured at monitoring sites. This compares to an estimate of 1.64 for an increase of 10.4 μg/m^3^ PM_10_ in our analysis for the whole study population and 1.23 for women, only. The higher spatial resolution of the EURAD-model (1 km grid vs. 8 km grid for the monitoring station) might have led to reduced exposure misclassification, thereby increasing slightly the point estimate in our study. Similarly, the small-scale residential exposure modeling we used might contribute to the higher point estimate in our study for PM_2.5_ (1.37 for an increase of 10 μg/m^3^ PM_2.5_) compared to a recent Canadian cohort study (RR of 1.11 for a 10 μg/m^3^ increase of PM_2.5_), which used satellite-based exposure modeling with a resolution of approximately 10 km × 10 km [18].

We observed similar effects of traffic and total PM when referring to the IQR which is not surprising as this compares the PM exposures for the within population contrast in exposure. In contrast, there is a stronger effect for traffic-specific PM than for total PM, when comparing estimates for the same increase in PM-mass concentration (i.e., for an increase of 1 μg/m^3^), pointing towards a higher toxicity of traffic-specific air pollution compared to that of total PM. In the Los Angeles Black Women Health Study (BWHS), no association was observed with total PM_2.5_ with the effect estimate being very unstable (incidence rate ratio of 1.63 per 10 μg/m^3^, 95 %-CI 0.78,3.44) [16], but a significant association was observed for NO_x_, an indicator for traffic related pollution. Similarly, the cohort of the Danish Cancer Society (DDCH) yielded an association of the traffic indicator NO_2_ with confirmed incident diabetes [17]. Also, the previous study in the Ruhr area, SALIA, has found a stronger relation with the traffic indicator NO_2_ than with PM [14].

Corroborating our results for the modeled traffic exposure, we also found a clear effect for living near a busy road (<100 m vs >200 m). A positive association of living less than 100 m from a busy road (>10,000 cars /day) was also found by Krämer et al. [14], though only in women with a low educational level. Puett et al. [15] found a relation with distance to the nearest road in the women cohort (Nurses Health Study) but not in the male cohort (Health Professionals Follow-up Study).

Although these previous results indicated that effect sizes for traffic-related exposures might be specifically important, none of the previous studies has allowed a direct comparison between traffic-specific PM and total PM. In fact, our work is the first study that can make a direct comparison between the effects and hence indirectly of the toxicity related to the same amount (here for 1 μg/m^3^) of PM-mass from traffic PM and from total PM that also contains several constituents which are not relevant for health such as e.g., sea salt [25]. Indeed our results indicate that in the investigated area, 1 μg/m^3^ of traffic-specific PM is more toxic than 1 μg/m^3^ total PM. This is well in line with reports of higher toxicity of diesel exhaust particles (for a review see [33]) the importance of which is enhanced by diesel motor vehicles being fairly common in Germany. Diesel engine exhaust has been related to increased inflammation of the airways [34] and a reduction of cardiovascular function [35] in healthy volunteers. Gasoline emissions, another important part of traffic related air pollution, have been shown to be related to vascular remodeling and vascular oxidative stress on ApoE^−/−^-mice [36]. A review of epidemiological studies also points to a higher toxicity of traffic-related sources in comparison to total PM and several other sources [37].

One of the mechanisms linking particulate matter exposure with metabolic disease is hypothesized to be an increase of systemic oxidative stress and inflammation e.g., in endothelial cells and macrophages [3]. In a mouse model, PM_2.5_ leads to an increase in TNF-α, IL-6, resistin and leptin consistent with a pro-inflammatory insulin resistant state [38]. PM-induced overactivity of the sympathetic nervous system may exacerbate inflammation-induced systemic insulin resistance [39].

A link of PM_10_ with insulin resistance (HOMA-R test) was found in a cross-sectional study on children (6-18 years) in Iran [40]. In line with this, a study on an elderly population in Taiwan showed a cross-sectional association of PM _2.5_ with glucose levels and HbA1c [41]. Whereas in these latter two countries air pollution levels are higher than those experienced in the US and Europe [42], Brook et al. [42] showed that also ambient levels encountered in US cities (<35 μg/m^3^ PM_2.5_) lead to alterations in insulin sensitivity: taking into account the exposure of the past five days, higher PM_2.5_ concentrations were linked to higher glucose levels and lower insulin sensitivity in 25 healthy volunteers [42]. A long-term effect at even lower concentrations (mean concentration <15 μg/m^3^) was recently observed by a prospective birth cohort of 397 children in Germany where an association of traffic-related air pollution at the birth address with insulin resistance at age 10 was reported [43]. Regarding an alternative pathway, Brook et al. hypothesized that autonomic imbalance favoring sympathetic activity may contribute to a short-term decrease in insulin sensitivity based on their observation of an association between insulin sensitivity and overall heart rate variability [42]. The latter has repeatedly been shown to be associated with PM exposure [3].

In our study, we found a higher effect in individuals with a high BMI and age ≥65 which could be in line with a role of systemic inflammation. Obesity is linked to higher levels of systemic inflammation [44] and therefore obese individuals could be more susceptible to additional stressors that act also via this pathway. Similarly, ageing has been shown to be related to subclinical systemic inflammation which is thought to reflect ageing of the immune system in presence of continuous antigenic exposure [45].

Interestingly, we found clearer and stronger results for the city-adjusted model which can be considered to be a more conservative approach as it is based only on the contrasts within the single cities. A similar observation was made in a study on cardiovascular events in 36 US metropolitan areas [46]: the observed effects for the within city contrasts (comparable to our main model) were stronger than the between city contrast and also larger than the effect from the model that did not distinguish between the two (comparable to our main model without city). As these authors point out, the “within-city” approach reduces concern over (uncontrolled) confounders that may vary between cities including unmeasured subjects characteristics and differences in air pollution mixtures.

A strength of our study is the prospective design in a large population-based sample with detailed information on risk factors. Sensitivity analyses yielded the same results when defining diabetes mellitus by self-reported disease only.

A major limitation of our study is the availability of only modeled values for traffic-specific PM_2.5_ and PM_10_. Different approaches have been applied in other studies to overcome this problem, such as using exposure indicators like black carbon or elemental carbon, which primarily result from combustion processes and are likely to reflect traffic exposure differences in highly urbanized areas with high traffic density. We applied a different approach by excluding traffic emissions in a scenario calculation of a chemistry transport and dispersion model. However, it is not possible to validate the model output at this time, since no method to identify exclusively traffic-generated particles exists up to now. Another limitation is that we could not account for the mobility of study participants. However, this is likely to increase imprecision of the exposure assessment and therefore introduce a bias towards the null. Also our sensitivity analysis on participants that have not moved during follow-up did not differ markedly from the main results. While the follow-up population did not differ markedly from the base population, there was a tendency for a higher drop out rate for unemployed individuals, pensioners and individuals with hypertension. These are characteristics that also occur more frequently among incident diabetics. Thus, if air pollution causes diabetes and if pollution exposure was on average higher among those individuals, our relative risks would actually underestimate the real risk.

## Conclusion

Long-term exposure to total PM increases type 2 diabetes risk in the general population. Traffic-specific PM seems to be specifically toxic on an equal mass basis. Future investigations should try to elucidate further the involved pathomechanisms e.g., by studying intermediates on the hypothesized pathways. Approaches to model air pollution exposure would benefit from the availability of emission inventories and measurements of chemical constituents of the PM mixture.

## Additional file

Additional file 1: Table S1. Distributions of particulate matter (PM_2.5_, PM_10_) in the study population. **Figure S1.** Relative risks (RR) for an increase of 1 μg/m^3^with 95 % confidence intervals from different adjustment sets.
