# Erratum to: Long-term exposure to fine particulate matter and incidence of type 2 diabetes mellitus in a cohort study: effects of total and traffic-specific air pollution

**DOI:** 10.1186/s12940-016-0128-x

**Published:** 2016-03-23

**Authors:** Gudrun Weinmayr, Frauke Hennig, Kateryna Fuks, Michael Nonnemacher, Hermann Jakobs, Stefan Möhlenkamp, Raimund Erbel, Karl-Heinz Jöckel, Barbara Hoffmann, Susanne Moebus, on behalf of the Heinz Nixdorf Recall Investigator Group

**Affiliations:** IUF - Leibniz Research Institute for Environmental Medicine, Düsseldorf, Germany; Medical School, Heinrich Heine University of Düsseldorf, Düsseldorf, Germany; Institute for Medical Informatics, Biometry and Epidemiology, University Hospital of Essen, University of Duisburg-Essen, Essen, Germany; Rhenish Institute for Environmental Research at the University of Cologne, Cologne, Germany; Department of Cardiology, West German Heart Centre of Essen, University of Duisburg-Essen, Essen, Germany

Unfortunately, the original version of this article [1] contained an error. The paragraph of the results starting with “The relative risks…” contained errors in the reported effect estimates and confidence intervals.

The paragraph read:

The relative risks from the unadjusted crude model and for the main model are shown in Table 2. When expressing RRs per IQR, exposure to total PM_10_ was related to an increase in type 2 diabetes incidence of 20 % (RR of 1.20, 95 %-CI: 1.01;1.31) in the main model. The corresponding RR for PM_2.5_ was 1.11 (95 %-CI: 0.99;1.23). For traffic-specific PM, the estimates for this measure of population distribution of exposures were similar with a RR of 1.11 (95 %-CI: 0.99;1.17) for PM10_TRA_ and a RR of 1.10 (0.99;1.23) for PM2.5_TRA_.

But it should have read:

The relative risks from the unadjusted crude model and for the main model are shown in Table 2. When expressing RRs per IQR, exposure to total PM_10_ was related to an increase in type 2 diabetes incidence of 20 % (RR of 1.20, 95 %-CI: 1.01;**1.42**) in the main model. The corresponding RR for PM_2.5_ was **1.08** (95 %-CI: **0.89;1.29**). For traffic- specific PM, the estimates for this measure of population distribution of exposures were similar with a RR of 1.11 (95 %-CI: 0.99;**1.23**) for PM10_TRA_ and a RR of 1.10 (0.99;1.23) for PM2.5_TRA_.
