# Supplementary material for: Long-term exposure to fine particulate matter and incidence of type 2 diabetes mellitus in a cohort study: effects of total and traffic-specific air pollution
Source: Environ Health. 2015 Jun 19;14:53. doi: 10.1186/s12940-015-0031-x (PMC4479324; doi:10.1186/s12940-015-0031-x)
Supplement: Additional file 1: Table S1. — Distributions of particulate matter (PM2.5, PM10) in the study population. Figure S1. Relative risks (RR) for an increase of 1 μg/m3with 95 % confidence intervals from different adjustment sets. [file 12940_2015_31_MOESM1_ESM.pdf]

Additional File 1

Long-term exposure to fine particulate matter and incidence of type 2 diabetes mellitus: Effects of total and traffic-specific air pollution

Gudrun Weinmayr[1,2], Frauke Hennig[1], Kateryna Fuks[1], Michael Nonnemacher[3], Hermann Jakobs[4], Stefan Möhlenkamp[5], Raimund Erbel[5], Karl-Heinz Jöckel[3], Barbara Hoffmann[1,2&], Susanne Moebus[3&] on behalf of the Heinz Nixdorf Recall Investigator Group

1 IUF - Leibniz Research Institute for Environmental Medicine, Düsseldorf

2 Medical School, Heinrich Heine University of Düsseldorf, Düsseldorf, Germany

3 Institute for Medical Informatics, Biometry and Epidemiology, University Hospital of Essen, University of Duisburg-Essen

4 Rhenish Institute for Environmental Research at the University of Cologne

5 Department of Cardiology, West German Heart Centre of Essen, University of Duisburg-Essen

& B.H. and S.M. contributed equally to last authorship

Address for correspondence:

Gudrun Weinmayr

E-Mail: gudrun.weinmayr@uni-duesseldorf.de, gudrun.weinmayr@uni-ulm.de

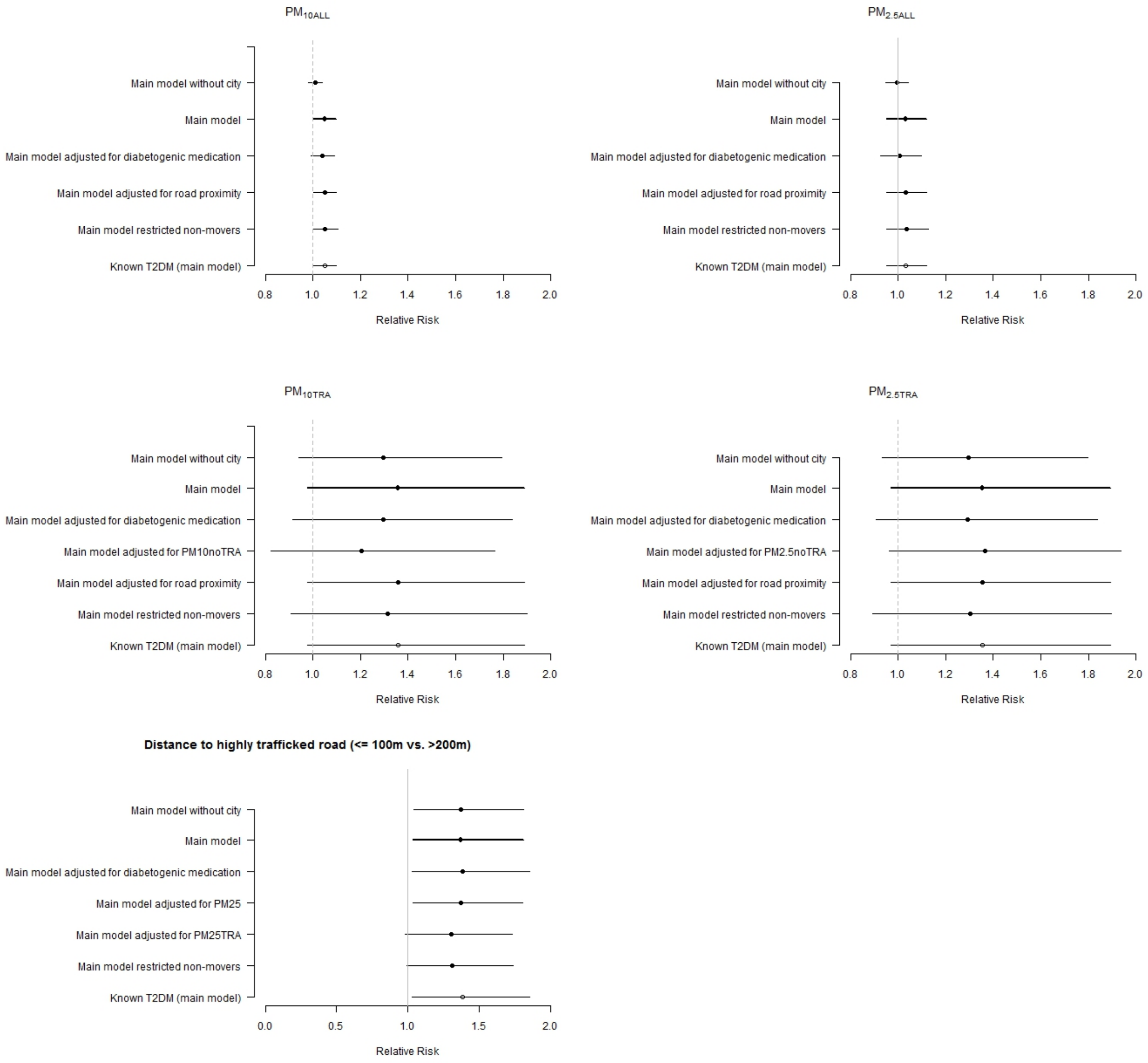

addFig.1. Relative risks (RR) for an increase of 1µg/$m^3$with 95% confidence intervals from different adjustment sets:
Main model: adjusted for sex, age, BMI, lifestyle, individual SES, neighborhood unemployment rate and city (N=3607)
Known T2DM: outcome based on self-reported physician diagnosed T2DM or intake of medication for T2DM (N=3372)
Main Model restricted to movers is based on 3006 participants, all other models (except Known T2DM) on 3607 participants.

addTable 1: Distributions of particulate matter ($PM_{2.5}$, PM_{10}) in the study population (N=3607).

-	Annual mean 2001-2002	Mean	SD	Min	Q1	Q3	Max	IQR	Correlation coefficient (Pearson)			
									PM_{10ALL}	PM_{10TRA}	$PM_{2.5ALL}$	$PM_{2.5TRA}$
PM_{10} (µg/m³)	Total (PM_{10ALL})	20.80	2.35	17.1	18.71	22.49	28.21	3.78	1			
	Traffic (PM_{10TRA})	0.83	0.24	0.24	0.64	0.97	1.69	0.33	0.52	1		
$PM_{2.5}$ (µg/m³)	Total ($PM_{2.5ALL}$)	16.74	1.44	14.16	15.54	17.83	20.95	2.29	0.88	0.39	1	
	Traffic ($PM_{2.5TRA}$)	0.83	0.24	0.26	0.65	0.97	1.66	0.32	0.51	1.00	0.38	1
Distance to major road [a] (m)		1,022	807	1	412	1,433	4,877	1,021	-0.26	-0.45	-0.12	-0.45

Q1 and Q3 = upper limit of 1st and 3rd quartile, respectively, IQR = interquartile range, a: major road is defined as a road with more than 26,062 vehicles/day
